# Supplementary material for: Influence of the luxR Regulatory Gene Dosage and Expression Level on the Sensitivity of the Whole-Cell Biosensor to Acyl-Homoserine Lactone
Source: Biosensors (Basel). 2021 May 23;11(6):166. doi: 10.3390/bios11060166 (PMC8224577; doi:10.3390/bios11060166)
Supplement: Supplementary file 1 [file biosensors-11-00166-s001.zip › biosensors-1209223-supplementary.pdf]

biosensors

Supplementary materials

Influence of the *luxR* regulatory gene dosage and expression level on the sensitivity of whole-cell biosensor to acyl-homoserine lactone

Sergey Bazhenov [1,2,3,*], Uliana Novoyatlova [1], Ekaterina Scheglova [1], Vadim Fomin [1], Svetlana Khrulnova [1,4], Olga Melkina [5], Vladimir Chistyakov [2] and Ilya Manukhov [1,3,6]

[1] Moscow Institute of Physics and Technology, 141701, Dolgoprudny, Russia; novoyatlova.us@phystech.edu (U.N.); scheglova.es@phystech.edu (E.S.); fomin.vv@phystech.edu (V.F.); khrulnovas@mail.ru (S.K.); manukhovi@mail.ru (I.M.)
[2] , Academy of Biology and Biotechnology, Southern Federal University, 344022, Rostov-on-Don, Russia; vladimirchi@sfedu.ru
[3] Faculty of Physics, HSE University, 109028 Moscow, Russia
[4] National Research Center for Hematology, 125167, Moscow, Russia
[5] State Research Institute of Genetics and Selection of Industrial Microorganisms of the National Research Center "Kurchatov Institute", 117545, Moscow, Russia; compleanno@mail.ru
[6] Federal Research Center of Biological Systems and Agro-technologies of RAS, 460000, Orenburg, Russia
* Correspondence: sergei.v.bazhenov@phystech.edu

Scheme of plasmids used in the study

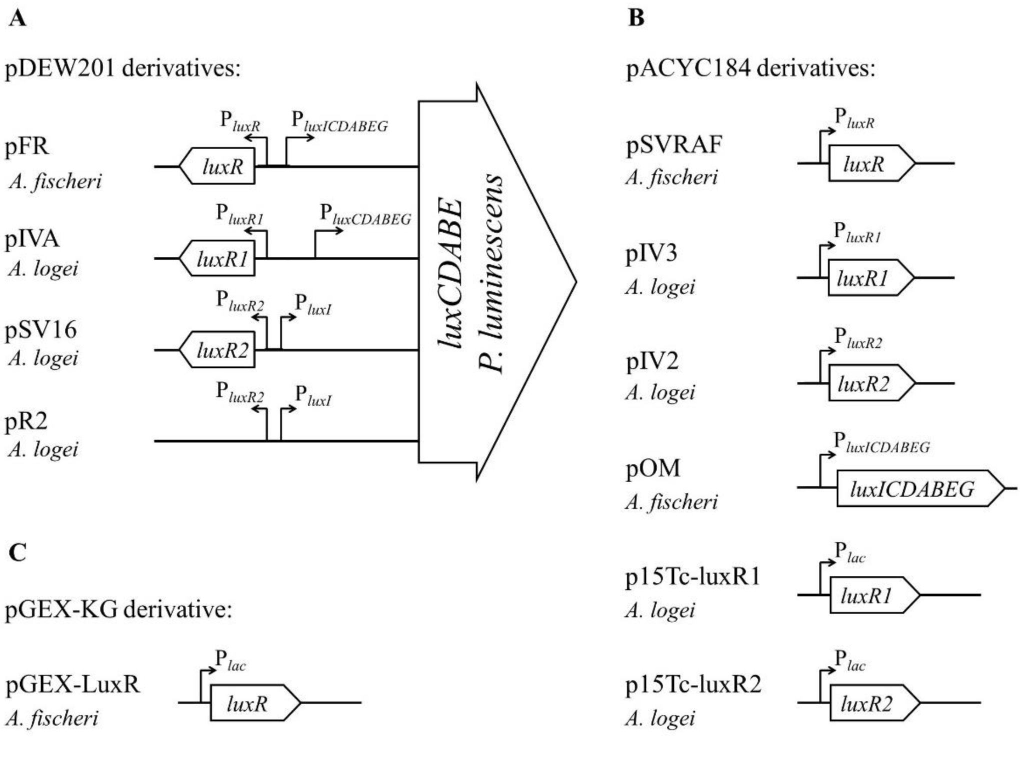

Figure S1. Scheme of key components of plasmids used in the study with specification of biosensor plasmids names and regulatory gene source bacteria species. **(A)** Promoters and genes being inserted in the pDEW201 promoter-probe vector with *luxCDABE Photorhabdus luminescens* genes. **(B)** The combinations of promoters and genes inserted in pACYC184 vector. **(C)** Promoter and *luxR* gene in pGEX-KG derivative.

Plasmid constructing

The p15Tc-lac vector was obtained by fusion of two PCR products with use of Gibson Assembly MasterMix (NEB, USA): the fragment with *lacI* and P_{tac} from pGexKG was amplified with Pf and Pr primers (Table S1), and the fragment with p15 ori and Tc^r gene was amplified with Vf and Vr primers from pACYC184.

The p15Tc-luxR1 plasmid was constructed on the base of the p15Tc-lac vector. The *luxR1* gene was amplified with R1f and R1r primers on a matrix of *A. logei* KCh1 gDNA, cloned into pAL2-T vector, and transferred to the p15Tc-lac vector at EcoRI site.

The p15Tc-luxR2 plasmid was constructed on the base of the p15Tc-lac vector. The *luxR2* gene was amplified with R2f and R2r primers on a matrix of *A. logei* KCh1 gDNA, and cloned into the p15Tc-lac vector at EcoRI/KpnI sites with use of Gibson Assembly.

The pR2 plasmid was constructed on the base of the pDEW201 vector. Promoter region of *luxI* gene (318 bp in length) was amplified with P2f and P2r primers on a matrix of *A. logei* KCh1 gDNA, cloned into pTZ57R/T vector at EcoRV site, and transferred to pDEW201 by KpnI/BamHI sites, ensuring that the *luxCDABE P. luminescens* genes are under control of P_{luxI} *A. logei* promoter.

Table S1. Oligonucleotides used for cloning promoters into promoter-probe vector and litR under P_{tac} promoter in this study.

Nucleotides, which are complementary to the matrix, are given in bold; 5′ region of most primers have adapters for cloning through Gibson Assembly or restriction/ligation.

**Primer**	**Sequence, 5′-3′**
Pf	AACGCGGAAG**CACACCGCATAAATTCCGACACCATC**
Pr	CACCCGGGTACC**TAGTATAGGGGACATGAATTCTGTT**
Vf	CTATACTAGGTACCC**CGGGTGATGCTGCCAACTTACTGA**
Vr	TGCGGTGTG**CTTCCGCGTTTCCAGACTTTACGA**
R1f	GAATTC**ATGAACTATATCGCTCCTCATGACAC**
R1r	**GATTATGTAAAAATAAATGAGGGAAGCCAGGT**
R2f	TAACAATTTCACACAGGAAACAGAATTC**ATGAGCTTATGCCTACAAGAACTAACTA**
R2r	GGCAGCATCACCCGGGTACC**GAAGTCCGACTGCGTTATTCTACA**
P2f	GGCATAAACTCATGTCGTTGTTGG
P2r	GTCATCCTGACCCCCTTTAATCTTT

Detailed results of *E. coli* MG1655 pVFR1 pSVRAF testing during the expeditions

In 2020, two research expeditions to the White and Azov Seas were conducted with the purpose of searching for acyl-homoserine lactones in environmental samples with the use of an AI-sensitive whole-cell biosensor based on *E. coli* MG1655 pVFR1 pSVRAF. List of samples is given in table S2.

Table S2. Samples collected and analyzed with *E. coli* MG1655 pVFR1 pSVRAF biosensor during the expedition to the Azov and to the White Seas.

**Azov Sea**			
**Sample**	**Date**	**Place**	Source
A1	02.08.20	Seashore of Taganrog Bay (47.285962 N., 39.172848 E.)	Slit under water
A3	02.08.20		water, 50m. from coast
A6	03.08.20		Slit from coast
A7	03.08.20		Water from a puddle in the mud on bank
TAR1	04.08.20	Seashore of Taganrog Bay, Taganrog embankment	Taranka fish

TAR2	04.08.20		Taranka fish
C1	04.08.20		crucian
C2	04.08.20		crucian
C3	04.08.20		crucian
C4	04.08.20		crucian
GA1	04.08.20		goby
GA2	04.08.20		goby
GA3	04.08.20		goby
GA4	04.08.20		goby
P1	04.08.20	Bank of the Don, Rostov embankment	goby
P2	04.08.20		goby
P3	04.08.20		goby
P4	04.08.20		goby
P5	04.08.20		goby
P6	04.08.20		goby
P7	04.08.20		goby
P8	04.08.20		goby
P9	04.08.20		goby
P10	04.08.20		goby
P11	04.08.20		goby
P12	04.08.20		goby
P13	04.08.20		goby
P14	04.08.20		goby
P15	04.08.20		goby
P16	04.08.20		goby
P17	04.08.20		goby
P18	04.08.20		goby
P19	04.08.20		goby
P20	04.08.20		goby
P21	04.08.20		goby
P22	04.08.20		goby
P23	04.08.20		goby
P24	04.08.20		goby
P25	04.08.20		goby
White Sea			
Sample	Date	Place	Source
COD1	22.07.20	Nilmoguba Bay (66.512362 N., 33.150385 E.)	codfish
COD2	22.07.20		codfish
S2	22.07.20		saffron cod
H1	22.07.20		herring
H2	22.07.20		herring
H3	22.07.20		herring
H4	22.07.20		herring
G1	22.07.20		goby
G2	22.07.20		goby
G3	22.07.20		goby
S3	23.07.20	Olenevskiy island (66.523172 N., 33.116968 E.)	Saffron cod
S4	23.07.20		Saffron cod

S5	23.07.20		Saffron cod
S6	23.07.20	Krivoy island (66.512310 N., 33.151998 E.)	Saffron cod
S7	23.07.20		Saffron cod
K1	23.07.20		whitespotted char
H5	23.07.20		herring
H6	23.07.20		herring
H7	23.07.20		herring

In the study of the intrinsic luminescence of the samples, it was revealed that without the addition of the bacterial luciferase substrate, decanal, luminescence was observed in sample P24 (30 rel.units) in sample H5 (870 rel. units), and with the addition of decanal (0.1%), in samples P7 and P24 (4000 and 1500 rel.units, respectively).

Then luminescence of biosensor cell culture, adding samples, was measured. *E. coli* MG1655 pSVRAF pVFR1 was used as a biosensor, in whose cells the expression of the *P. luminescens luxCDABE* genes are regulated by the *A. fischeri* LuxR protein with an increased *luxR* gene dosage. The cell culture of the biosensor strain was grown in a liquid LB medium, then divided into 200 µl aliquots in test tubes, to which liquid fractions and suspensions of the collected samples were added in a volume of 20 µl each. After one, two, and three hours, luminescence of each sample was measured. The results are given in Figure S2.

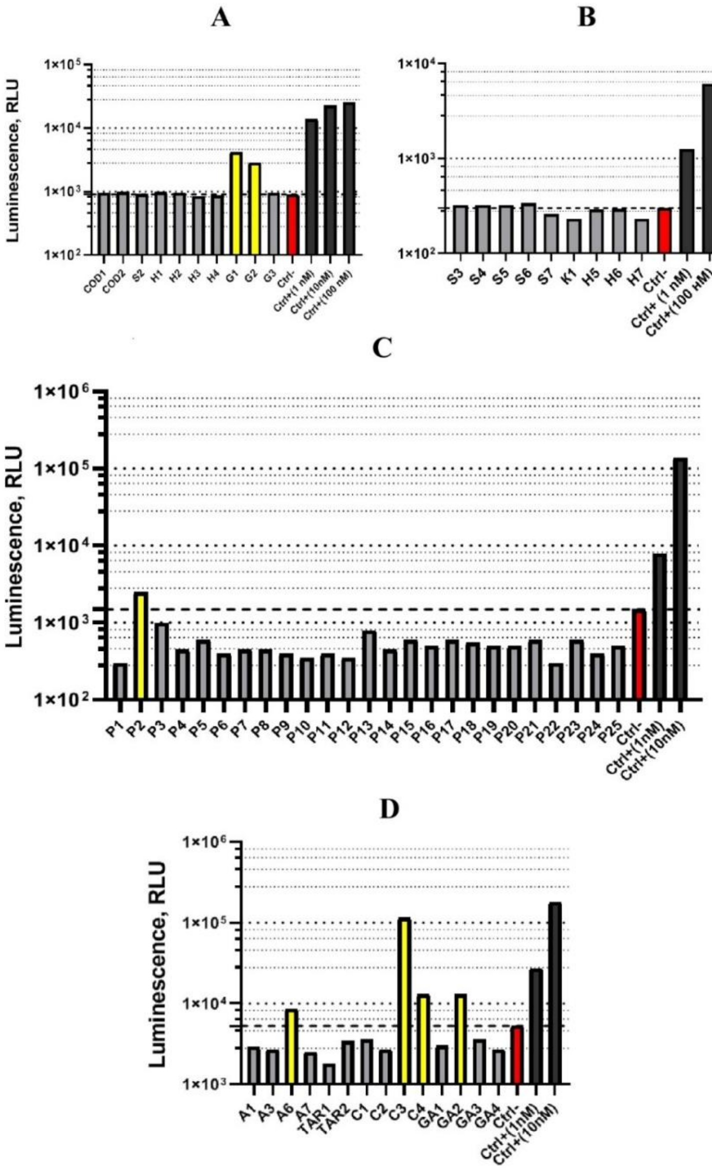

Figure S2. The luminescence of biosensor cell culture supplemented with 20 μL of environmental samples (listed in table S1). Ctrl- is a negative control: biosensor cell culture without additives. Ctrl+(1nM), Ctrl+(10 nM) and Ctrl+(100 nM) are positive controls: biosensor cell culture supplemented AI at the final concentrations 1 nM, 10 nM and 100 nM, respectively. Bars in yellow are samples that caused an increase in luminescence. (A) Series of measurements taken at the Azov Sea expedition on the 4.08.2020. (B) Series of measurements taken at the Azov Sea expedition on the 4.08.2020 (C) Series of measurements taken at the White Sea expedition on the 22.07.2020 (D) Series of measurements taken at the White Sea expedition on the 23.07.2020.

It was found that samples A6, C3, C4, GA2, P2, G1, and G2 caused an increase in the luminescence of cells of the biosensor strain *E. coli* MG1655 pVFR1 pSVRAF. Samples GA2, P2, and A6 were seeded on a solid media SWT. Three strains, BCh1, BCh2, and BCh3, were isolated from obtained cultures, respectively, by ability to induce luminescence of biosensor cells while being located near each other. By sequencing the 16S rRNA gene with a pair of primers, 5′-CGTGCCAGCAGCCGCGGTAATA-3′ and 5′-GTGTGTACAAGGCCCGGGAACG-3′, it was identified that strains BCh1, BCh2, and BCh3 belong to *Aeromonas veronii* or *Aeromonas hydrophila*.
