# Supplementary material for: Influence of the luxR Regulatory Gene Dosage and Expression Level on the Sensitivity of the Whole-Cell Biosensor to Acyl-Homoserine Lactone
Source: Biosensors (Basel). 2021 May 23;11(6):166. doi: 10.3390/bios11060166 (PMC8224577; doi:10.3390/bios11060166)
Supplement: Supplementary file 1 [file biosensors-11-00166-s001.zip › biosensors-1209223.pdf]

biosensors

Article

Influence of the *luxR* Regulatory Gene Dosage and Expression Level on the Sensitivity of the Whole-Cell Biosensor to Acyl-Homoserine Lactone

Sergey Bazhenov [1,2,3,*], Uliana Novoyatlova [1], Ekaterina Scheglova [1], Vadim Fomin [1], Svetlana Khrulnova [1,4], Olga Melkina [5], Vladimir Chistyakov [2] and Ilya Manukhov [1,3,6]

1 Moscow Institute of Physics and Technology, 141701 Dolgoprudny, Russia; novoyatlova.us@phystech.edu (U.N.); scheglova.es@phystech.edu (E.S.); fomin.vv@phystech.edu (V.F.); khrulnovas@mail.ru (S.K.); manukhovi@mail.ru (I.M.)
2 Academy of Biology and Biotechnology, Southern Federal University, 344022 Rostov-on-Don, Russia; vladimirchi@sfedu.ru
3 Faculty of Physics, HSE University, 109028 Moscow, Russia
4 National Research Center for Hematology, 125167 Moscow, Russia
5 State Research Institute of Genetics and Selection of Industrial Microorganisms of the National Research Center "Kurchatov Institute", 117545 Moscow, Russia; compleanno@mail.ru
6 Federal Research Center of Biological Systems and Agro-technologies of RAS, 460000 Orenburg, Russia
* Correspondence: sergei.v.bazhenov@phystech.edu

check for updates

Citation: Bazhenov, S.; Novoyatlova, U.; Scheglova, E.; Fomin, V.; Khrulnova, S.; Melkina, O.; Chistyakov, V.; Manukhov, I. Influence of the *luxR* Regulatory Gene Dosage and Expression Level on the Sensitivity of the Whole-Cell Biosensor to Acyl-Homoserine Lactone. *Biosensors* **2021**, *11*, 166. https://doi.org/10.3390/bios11060166

Received: 19 April 2021
Accepted: 20 May 2021
Published: 23 May 2021

Publisher's Note: MDPI stays neutral with regard to jurisdictional claims in published maps and institutional affiliations.

Copyright: © 2021 by the authors. Licensee MDPI, Basel, Switzerland. This article is an open access article distributed under the terms and conditions of the Creative Commons Attribution (CC BY) license (https://creativecommons.org/licenses/by/4.0/).

Abstract: *Aliivibrio fischeri* LuxR and *Aliivibrio logei* LuxR1 and LuxR2 regulatory proteins are quorum sensing transcriptional (QS) activators, inducing promoters of *luxICDABEG* genes in the presence of an autoinducer (3-oxo-hexanoyl-l-homoserine lactone). In the *Aliivibrio* cells, *luxR* genes are regulated by HNS, CRP, LitR, etc. Here we investigated the role of the *luxR* expression level in LuxI/R QS system functionality and improved the whole-cell biosensor for autoinducer detection. *Escherichia coli*-based bacterial *lux*-biosensors were used, in which *Photorhabdus luminescens luxCDABE* genes were controlled by LuxR-dependent promoters and *luxR*, *luxR1*, or *luxR2* regulatory genes. We varied either the dosage of the regulatory gene in the cells using additional plasmids, or the level of the regulatory gene expression using the lactose operon promoter. It was shown that an increase in expression level, as well as dosage of the regulatory gene in biosensor cells, leads to an increase in sensitivity (the threshold concentration of AI is reduced by one order of magnitude) and to a two to threefold reduction in response time. The best parameters were obtained for a biosensor with an increased dosage of *luxR A. fischeri* (sensitivity to 3-oxo-hexanoyl-l-homoserine lactone reached 30–100 pM).

Keywords: whole-cell biosensor; LuxR; autoinducer; quorum sensing

1. Introduction

Quorum Sensing (QS) is a genetic mechanism enabling bacteria to determine population density through the exchange of specific signaling molecules, called autoinducers (AI). The first QS system discovered and described in detail was the LuxI/LuxR system of marine luminescent bacteria *Aliivibrio fischeri* [1]. LuxI produces AI, predominantly a 3-oxo-hexanoyl-l-homoserine lactone (3OC6-HSL), which passively penetrates the cell membrane and serves for signal transmission between cells [2]. LuxR is a regulatory protein, an AI-sensitive transcription activator. At a sufficient concentration of AI in the medium, the LuxR-AI complex is formed and, by binding to the lux-box in the promoter region, it induces the transcription of *luxICDABEG* genes [3]. In the closely related psychrophilic bacteria *Aliivibrio logei* and *Aliivibrio salmonicida* the LuxI/LuxR QS system differs from that in *A. fischeri*: the regulatory gene is represented by two homologues: *luxR1* and *luxR2* [4–6].

Bacterial QS systems regulate biofilm formation, synthesis of virulence factors, bioluminescence, and antibiotic resistance [7]. Detection of signaling molecules can enable early detection of a pathogen infection. The search for autoinducers is predominantly carried out using mass spectrometry [8–10]; however, this approach requires rather laborious sample preparation and expensive equipment. Whole-cell bacterial biosensors sensitive to AI are an alternative [11]; these can be used not only to search for AI and AI-synthesizing microorganisms [12,13], but also to investigate compounds that could block QS systems [14,15].

There are different reporter systems used to create whole-cell biosensors: fluorescent, luminescent, or colorimetric [16]. The first whole-cell biosensor sensitive to AI was constructed on the base of the *A. fischeri* LuxI/LuxR system and *lacZ* reporter gene [17]. Additionally, AI-sensitive biosensors have been developed with *luxCDABE* reporter genes, which provide a high rate of biosensor response [18], and the *gfp-mut3* reporter gene, which provides a decrease in the fluorescent signal following the disappearance of AI in the medium [12]. All aforementioned whole-cell biosensors based on *A. fischeri* LuxR are capable of detecting 3OC6-HSL at concentrations above 1 nM.

The process of the AI-dependent activation of transcription by a LuxR-type protein can be described using dissociation equations as two sequential phenomena: the formation of the LuxR-AI complex, and its binding to the *lux*-box in the promoter region [19]. This model predicts an increase in sensitivity of the system to low concentrations of AI with a concentration increase in the LuxR protein in the cell. Such an effect was described for the TraR protein [20] when comparing *Escherichia coli*-based biosensors with the *Agrobacterium tumefacienstraR* gene under control of P_{tetR} and P_{T7} promoters. As expression of regulatory *luxR* gene in *Aliivibrio sp.* cells depends on many intracellular factors (HNS, CRP, LitR, GroEL/ES, Lon, etc.) [21–24], a natural question to be asked is how the expression level of different *luxR* genes influence the corresponding QS systems. Though it is expected that QS sensitivity would be dependent on LuxR concentration, this has not been demonstrated in any prior experiments, and the enhanced *luxR* expression has had no practical use to date. In this work, we investigated the sensitivity of LuxR-based biosensors to AI depending on the regulatory gene dosage and the level of its transcription involving the following three regulatory genes: *luxR1* and *luxR2 A. logei*, and *luxR A. fischeri*.

2. Materials and Methods

2.1. Bacterial Strains and Plasmids

Bacterial strains and plasmids used in the study are listed in Table 1. Primers and detailed descriptions of plasmids constructed in this study are presented in the supplementary file.

To study the effect of the dosage of the *A. fischeri luxR* and *A. logei luxR1* and *luxR2* genes on the sensitivity of QS systems to AI, we compared pairs of *E. coli* MG1655 based biosensor strains carrying the following plasmids or combinations (enhanced *luxR* dosage due to the presence of the gene in both introduced plasmids): pFR or (pFR, pSVRAF), pIVA or (pIVA, pIV3), and pSV16 or (pSV16, pIV2) (Table 1). To investigate the role of their expression level, regulatory genes were placed under control of P_{lac}; *E. coli* MG1655 cells were transformed with the following plasmid combinations: pOM and pGEX-LuxR (*luxR A. fischeri* under P_{lac}); pR2 and p15Tc-luxR2 (*luxR2 A. logei* under P_{lac}); and pR2 and p15Tc-luxR1 (*luxR1 A. logei* under P_{lac}). As a negative control, cells insensitive to AI and IPTG with only the pR2 plasmid, without any *luxR* gene present, were used.

Table 1. Bacterial strains and plasmids used in the study.

**Strain**	**Genotype**	**Source**
E. coli MG1655	F-, λ-, ilvG-, rfb-50, rph-1	[25]
Aeromonas spp. BCh1, BCh2, BCh3, and BCh4	wild type	fish intestine, Sea of Azov, Taganrog Bay
Plasmid	Description	Source
pIVA	pDEW201 promoter-probe vector with *luxR1 A. logei* under control of P_{luxR1} *A. logei* and *luxCDABE P. luminescens* under control of $P_{luxCDABEG}$ *A. logei*, Ap^r	[22]
pSV16	pDEW201, *luxR2 A. logei* under control of P_{luxR2} *A. logei*, *luxCDABE P. luminescens* under control of P_{luxI} *A. logei*	[26]
pR2	pDEW201, *luxCDABE P. luminescens* under control of P_{luxI} *A. logei*	This study
pVFR1	pDEW201, *luxR A. fischeri* under control of P_{luxR} *A. fischeri*, *luxCDABE P. luminescens* under control of $P_{luxICDABEG}$ *A. fischeri*	[27]
pIV2	pACYC184, *luxR2 A. logei* under control of P_{luxR2} inserted into BamHI site, Cm^r	[22]
pIV3	pACYC184, *luxR1 A. logei* under control of P_{luxR1} inserted into BamHI site, Cm^r	[22]
pSVRAF	pACYC184, *luxR2 A. logei* under control of P_{luxR} inserted into BamHI site, Cm^r	[23]
pGEX-luxR	pGEX-KG vector containing the *luxR A. fischeri* gene under P_{tac} promoter; Ap^r	[27]
pOM	pACYC184 with a BamHI/NruI fragment of *A. fischeri* DNA from pF1 (*luxICDABEG* under the $P_{luxICDABEG}$ promoter and *lux*-regulatory DNA between *luxR* and *luxI* [without *luxR*]); Cm^r	[28]
p15Tc-lac	Gene-expression vector obtained by the ligation of the pGex-KG plasmid fragment (*lacI* and P_{lac}) with pACYC184 plasmid fragment (ori p15A and TcR), Tc^r	This study
p15Tc-luxR1	p15Tc-lac, *luxR1 A. logei* under control of P_{lac}, Tc^r *luxR1* was isolated from pIVA and cloned into p15Tc-lac	This study
p15Tc-luxR2	p15Tc-lac, *luxR2 A. logei* under control of P_{lac}, Tc^r *luxR2* was isolated from pSV16 and cloned into p15Tc-lac	This study

2.2. Culture Medium and Growth Conditions

E. coli cultures were grown in Lysogeny Broth (LB) at 37 °C. The LB medium was composed of 1% tryptone, 0.5% yeast extract, and 1% NaCl. The medium was supplemented with 100 µg/mL ampicillin, 20 µg/mL chloramphenicol, or 20 µg/mL tetracycline. Bacteria were grown on a solid medium (LB + 1.5% agar). Overnight cultures were used to inoculate liquid LB. The resulting cultures were grown with continuous agitation. The optical density (OD) of cell suspensions was measured with a KFK-3 photometer (ZOMP, Russia). For experiments on determining the sensitivity of biosensors, *E. coli* MG1655 cells carrying hybrid plasmids or combinations thereof were grown in liquid LB at 37 °C to OD of approximately 0.1, then placed into a microwell plate for luminescence measurements. For the induction of P_{lac}, IPTG was added to the final concentration of 0.1 mM immediately prior to the addition of AI. AI was added to the final concentrations of 10 pM to 1 mM with tenfold dilutions. For cultivation of marine bacteria, the SWT medium was used (w/v, %: tryptone 0.5, yeast extract 0.25, sea salt 1.5, glycerol 0.3, agar (for solid medium) 1.5).

2.3. Measurement of Bioluminescence

Bioluminescence intensities of 200 µL portions of cell culture were measured in 96 well plates using SynergyHT (Biotek Instruments, Winooski, VT, USA), or in capeless micro-

tubes using Biotox-7BM (BioPhysTech, Russia), which is 100 times more sensitive than SynergyHT. Luminescence values were expressed in relative light units (RLU), specific to each luminometer.

2.4. Determination of the Minimum Detectable Concentration

Minimum detectable concentration of AI is minimum concentration, which statistically significantly induces luminescence of biosensor cells (mean of induction factor is above 1 with confidence level of 0.05, one-way t-test, 3 independent biological replicates).

2.5. Data Processing

Error bars on graphics correspond to standard deviations calculated for 3 replicates. Luminescence induction factor (LIF) was calculated by dividing the luminescence of the cell culture portion with added AI (Lum(AI)) by the luminescence of the same cell culture portion without AI (Lum(Ctrl)).

$$\text{LIF} = \frac{\text{Lum(AI)}}{\text{Lum(Ctrl)}} \quad (1)$$

2.6. DNA Manipulation

Plasmid DNA was isolated with use of GeneJET Plasmid Miniprep Kit (Thermo Scientific, Waltham, MA, USA). Cell transformation with hybrid plasmids, agarose gel electrophoresis, and isolation of DNA fragments from agarose gel were performed according to [29]. Restriction and ligation reactions were carried out using enzymes from Promega (Madison, WI, USA).

2.7. Chemicals

AI—3-oxo-hexanoyl-l-homoserine lactone (3OC6-HSL) was supplied by Sigma (USA). IPTG—Isopropyl β-D-thiogalactopyranoside solution (Sigma, St. Louis, MO, USA).

3. Results

3.1. Influence of the Regulatory Gene Dosage on the Biosensor Sensitivity to AI

For each of the *luxR*, *luxR1*, and *luxR2* genes, the effect on the nearest LuxR-regulated promoter of the *lux* operon (e.g., P_{luxI} for *luxR2 A. logei*) was investigated. The graphs of dependence of the increase in luminescence on the concentration of AI in the medium are shown in Figure 1. The luminescence measurement was carried out 2 h after the AI addition.

We observed that a dosage increase in *luxR A. fischeri* and *luxR2 A. logei* genes (and likely of LuxR and LuxR2 protein concentrations in cells) leads to an increase in the sensitivity of biosensor cells and provides a decrease in the minimum detectable concentration of AI by approximately one order of magnitude.

An increase in the dosage of the *A. logei luxR1* gene did not lead to a significant difference in the properties of the corresponding biosensors, which may be associated with the regulation of *luxR1* itself.

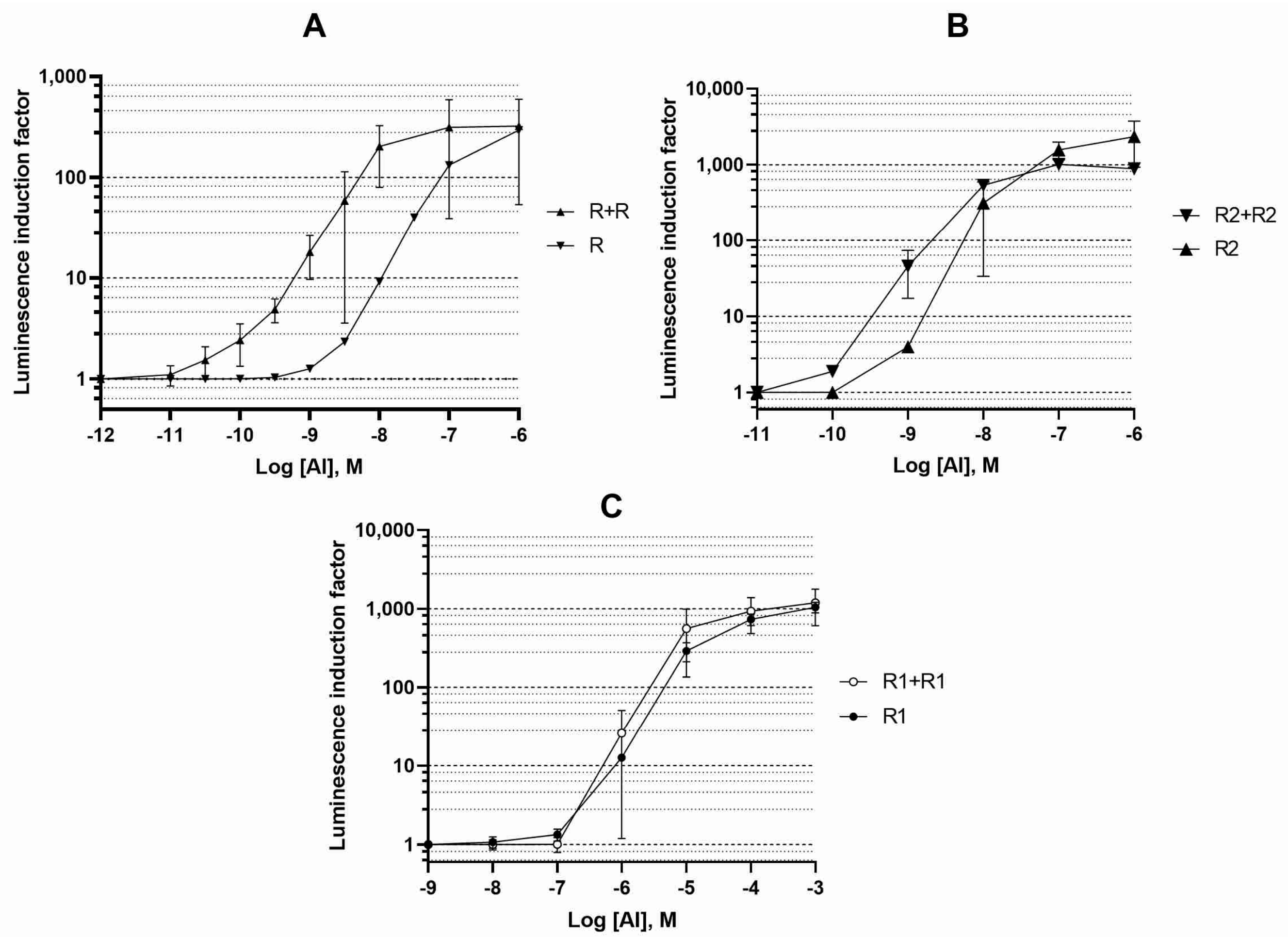

Figure 1. Dependence of the luminescence induction factor on AI concentration. The cultures of biosensor strains *E. coli* with a single copy of a regulatory gene on the pDEW201 vector (R for *luxR A. fischeri*, R1 and R2 for *luxR1* and *luxR2 A. logei*), and two copies of a regulatory gene on pDEW201 and pACYC184 vectors (R + R, R1 + R1, and R2 + R2, respectively) were used. The graphs show the average of three independent replicates.

3.2. *Influence of the luxR Genes Expression Level on the Sensitivity of the Biosensor*

In view of the absence of any effect from increasing the *luxR1* gene dosage on the QS system sensitivity to the AI, the effect of the enhanced expression level of the *luxR1* gene under control of a promoter unrelated to the QS system was investigated. The possibility of improving biosensor sensitivity to AI was tested for *luxR*, *luxR1*, and *luxR2* genes. Sensitivity of *E. coli* strains, which carry *luxR*, *luxR1*, or *luxR2* under control of P_{lac} to AI, was compared (Figure 2) with that of strains with a different regulatory gene (*luxR*s) dosage, which were described in the previous part.

The graph shows that the addition of 1 mM IPTG to the culture of *E. coli* MG1655 (pR2, p15Tc-luxR1) cells leads to an increase in the sensitivity and makes it possible to detect approximately 10 times lower concentrations of AI. Without IPTG addition, the minimum detectable concentration of AI for *E. coli* MG1655 (pR2, p15Tc-luxR1) cells is 1 µM; the same cells supplemented with IPTG are able to detect the AI at concentrations of 100 nM and above. For *luxR A. fischeri* and *luxR2 A. logei* the effect is the opposite—an enhancement of the regulatory gene expression by IPTG leads to a decrease in both the sensitivity to AI and the AI-dependent induction amplitude of the biosensor. We assume this indicates the existence of some optimal regulatory gene dosage and expression level (and, consequently, the regulatory protein concentration in the cell) for the AI detection.

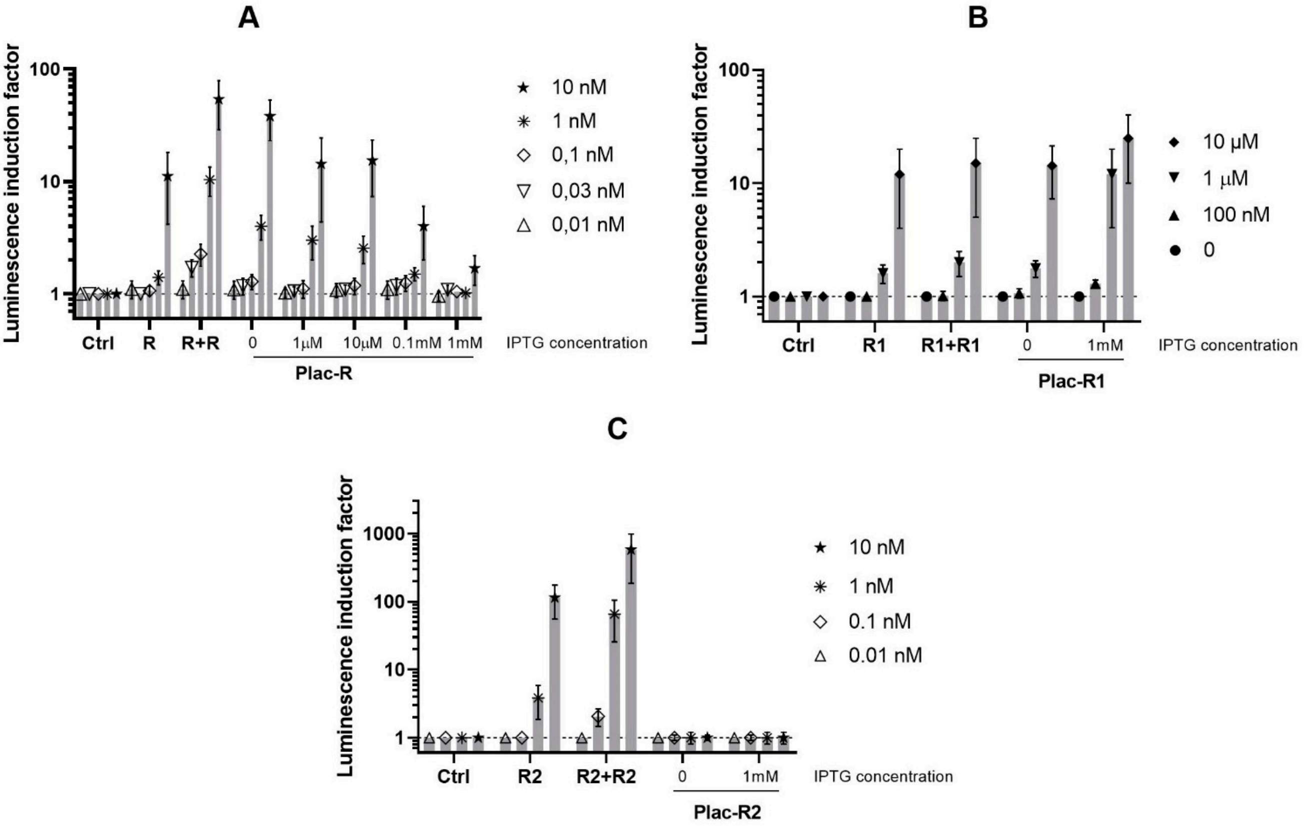

Figure 2. AI-dependent induction of luminescence of biosensor cells *E. coli* MG1655 with *luxCDABE P. luminescence* under control of LuxR-inducible promoter and *luxR A. fischeri*, *luxR1*, or *luxR2 A. logei.* Curves show the dependence of induction by specific AI concentration on regulatory gene dosage and its expression level (regulated with IPTG). The following plasmids were used: (**A**) pR2 (Ctrl, negative control without any *luxR* gene), pVFR1 (R, single *luxR A. fischeri* copy on pDEW201 vector), pVFR1 and pSVRAF (R + R, additional copy of *luxR A. fischeri* on pACYC184 vector), or pOM and pGEX-LuxR (Plac-R, *luxR A. fischeri* under P_{lac}); (**B**) pR2 (Ctrl), pIVA (R1, single *luxR1 A. logei* copy on pDEW201), pIVA and pIV3 (R1 + R1, additional copy of *luxR1 A. logei* on pACYC184), pR2 and p15Tc-luxR1 (Plac-R1, *luxR1 A. logei* under P_{lac}); (**C**) pR2 (Ctrl), pSV16 (R2, single *luxR2 A. logei* copy on pDEW201), pSV16 and pIV2 (R2 + R2, additional copy of *luxR2 A. logei* on pACYC184), or pR2 and p15Tc-luxR2 (Plac-R2, *luxR2 A. logei* under P_{lac}). The graphs show the average of three independent replicates.

3.3. Influence of the luxR Dosage and Expression Level on the Base Luminescence of Biosensor Cells

During the experiments with a set of constructed biosensors the variation of base luminescence—luminescence intensity of cell culture in absence of AI in the medium—was obtained. A comparison of background transcription (by luminescence) of LuxR-dependent promoters in the absence of AI in dependence on both the *A. fischeri luxR* and *A. logei luxR1* and *luxR2* genes dosage, and their expression level, is shown in Figure 3.

An increase in the dosage, or an increase in the expression level of each of the investigated *luxR* genes, is accompanied by an increase in the biosensor cells background luminescence value.

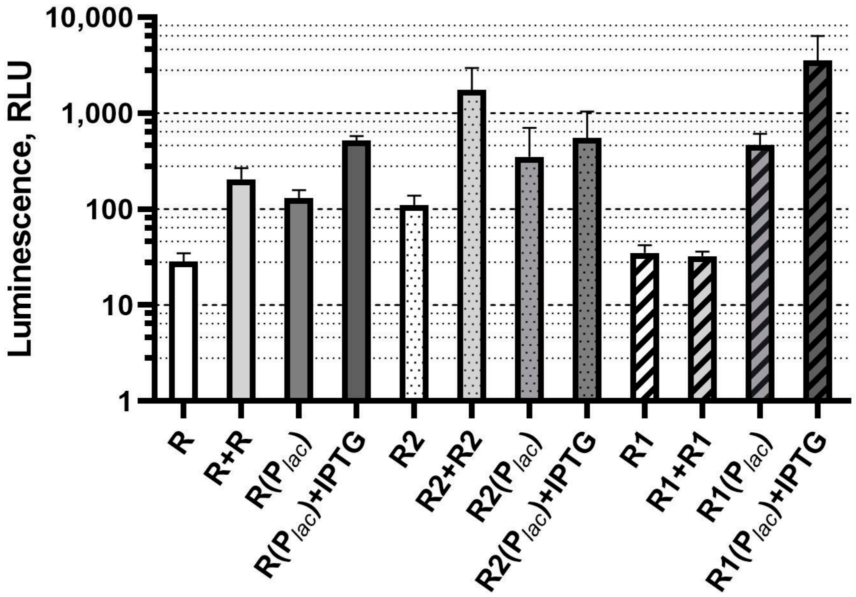

Figure 3. Dependence of base luminescence of LuxR-based biosensors on regulatory gene dosage and its expression level (without AI addition). Biosensor strains and corresponding signatures are the same as in previous figures. Graph shows averages of three independent replicates.

3.4. Influence of the Dosage and Expression Level of luxR Genes on the Biosensor Response Time

For variants of biosensors with the greatest effects from enhancing the regulatory gene dosage (*luxR A. fischeri*), and inducing regulatory gene expression level (*luxR1 A. logei*), characteristic luminescence kinetic curves are given in Figure 4. For biosensors with *luxR A. fischeri* (plasmid pVFR1), the kinetics of response to 10 nM AI is compared depending on the dose of the *luxR* gene, i.e., with and without the additional pSVRAF plasmid (Figure 4A). For the *A. logei luxR1*-based biosensor (a combination of pR2 and p15Tc-luxR1 plasmids), a comparison of the response kinetics with and without 1 mM IPTG is shown, i.e., at different levels of expression of the AI-sensitive regulator *luxR1* (Figure 4B).

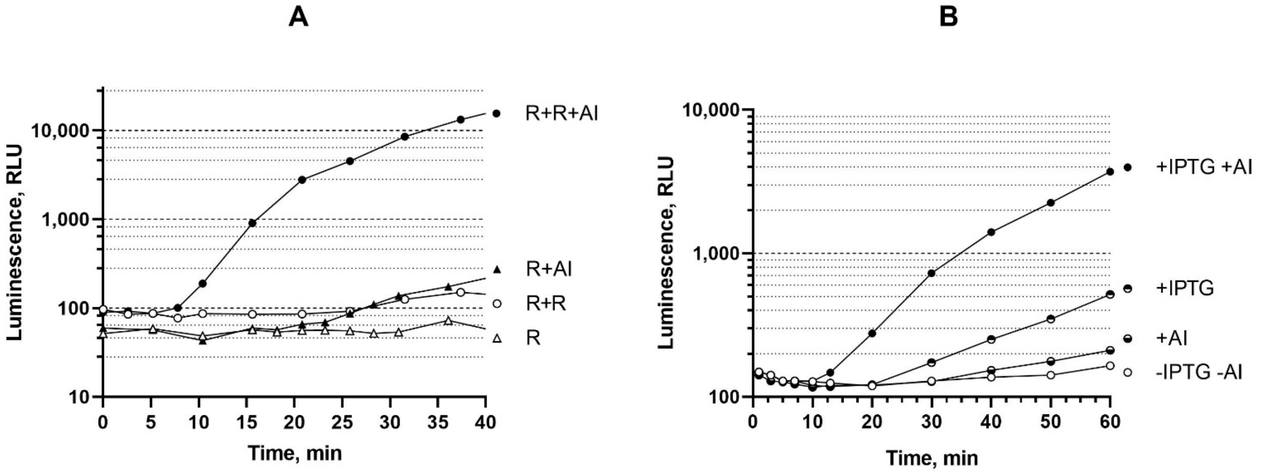

Figure 4. (**A**) Time-dependent luminescence of *E. coli* MG1655 cells carrying plasmid pVFR1 (R) and a combination of plasmids pVFR1 pSVRAF (R + R) after addition of AI to a final concentration of 10 nM. (**B**) Time-dependent luminescence of *E. coli* MG1655 (pR2, p15Tc luxR1) cells after the addition of 1 mM IPTG and/or 1 µM AI. Curves show dependencies for a single experiment which are characteristic for a raw of three independent replicates.

An increase in the dose of the *A. fischeri luxR* gene in biosensor cells leads to a response time reduction from 25 min to 7–10 min. There is significant difference between *luxR*, *luxR1*,

and *luxR2*-based biosensors: enhancement of *luxR1* expression with IPTG significantly improves sensitivity of the biosensor, while *luxR* and *luxR2*-based biosensors are much better in the case of the enhanced regulatory gene dosage. In terms of rate of response, extra copies of *luxR* and *luxR2* genes have nearly the same effect; in both cases time of response shortens to approximately 8 min. Similarly, the induction of the *luxR1* gene expression in the cells of the biosensor strain *E. coli* MG1655 pR2 p15Tc-luxR1 led to a threefold response reduction. Thus, an increase in the dosage of a regulatory gene, as well as an increase in the level of its expression with the help of an inducible promoter, could lead not only to an increase in the sensitivity of the biosensor, but also to a shorter response time.

3.5. Testing the Lux-Biosensor E. coli MG1655 pVFR1 pSVRAF in Expedition Conditions

The biosensor strain of *E. coli* MG1655 pVFR1 pSVRAF, which possesses the *luxR A. fischeri* gene at an increased dosage and *luxCDABE P. luminescens* genes under control of the *A. fischeri* $P_{luxICDABEG}$ promoter, was tested in expeditions to the White and Azov Seas, namely with samples of seawater, sea salt, and the intestinal contents of various fishes. This biosensor strain makes it possible to reliably detect the presence of 3OC6-HSL in the medium at concentrations of approximately 0.1 nM and higher. Minimum detectable concentration is about 0.03 nM, but samples are diluted during the measurement process with cell culture. The addition of a sample to the biosensor cell culture at a ratio of 1:1 or more makes results unstable. As a reference, the maximum achievable AI concentration during the cultivation of *A. salmonicida* and *A. logei* cells is 10 µM [8] and the concentration of AI, which significantly affects *A. logei* cells luminescence, is 100 nM and above (unpublished data). Portions of samples of either 2 or 20 µL were introduced into cuvettes with 200 µL of fresh culture of the biosensor strain grown in liquid LB to OD ~0.1. Furthermore, bioluminescence of the cultures with samples added was measured on a Biotox-7BM device. As a positive control, 3OC6-HSL in concentrations of 1 to 100 nM was used. On average, for 1 out of 10 samples of the intestinal content of fishes, an induction of luminescence of biosensor cells was observed. By magnitude, it was comparable to that with an addition of 3OC6-HSL at concentrations of 1–10 nM (see Supplementary Materials). Bacterial strains BCh1, BCh2, BCh3, and BCh4 were isolated from AI-containing samples and tested for their ability to synthesize AI by plating on one plate with the MG1655 pVFR1 pSVRAF biosensor strain (Figure 5). Near to the strains synthesizing AI, luminescence of biosensor cells visible to the naked eye were observed. A sequence analysis of 16S rRNA genes showed that isolated AI-synthesizing strains belong to the *Aeromonas veronii* or *Aeromonas hydrophila* species, for which the presence of type I QS system is known [30].

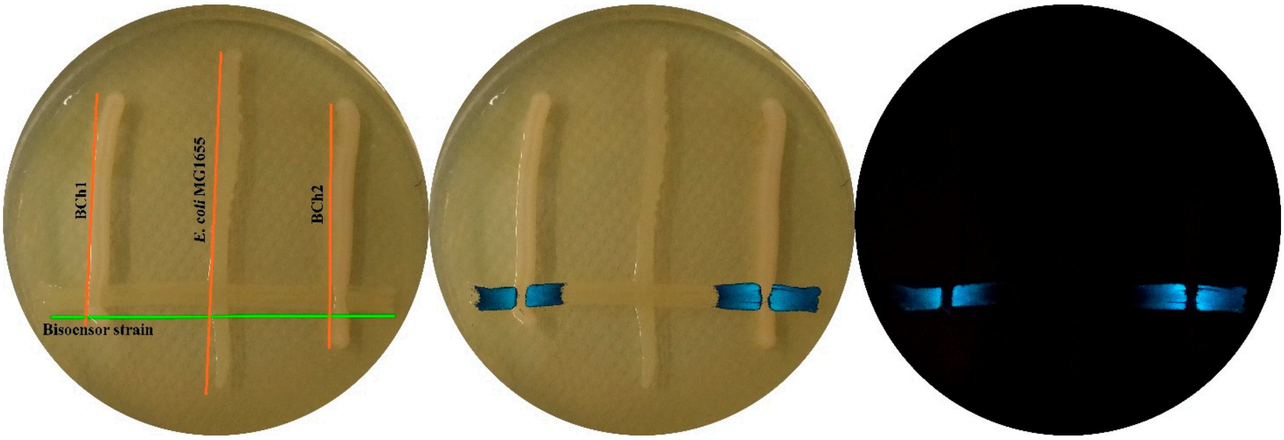

Figure 5. Photographs of Petri dishes with isolated strains and biosensor cells. Vertical line—*E. coli* MG1655 (as negative control) and isolated environmental bacterial strains BCh1 and BCh2; horizontal—biosensor cells of *E. coli* MG1655 pVFR1 pSVRAF sensitive to 3OC6-HSL. On the left—photograph under ambient light with annotation; on the right—in the dark, in the image in the middle an overlapping induced luminescence on photographs of Petri dishes under ambient light is shown.

4. Discussion

The resulting *E. coli* MG1655 pVFR1 pSVRAF biosensor surpasses previously created analogs in sensitivity to 3OC6-HSL due to the increased dosage of the regulatory gene (the minimum detectable concentration is 0.1 nM versus 1 nM and 5 nM previously [5,12]). These concentrations are close to the limitation determined by the cell volume and ability of LuxR, and the cell to bind and accumulate AI. An average *E. coli* cell volume is about 1 μm^3 [31]. When the AI concentration is 1 nM, and the AI molecules are evenly distributed in the medium, only one AI molecule can be detected in the volume of one cell. For the formation of the LuxR dimer and its association with DNA, at least two AI molecules are required. At the same time, the minimum detectable AI concentration for our biosensor is one AI molecule per 10 cell volumes. Thus, at extremely low concentrations of AI, the activation of biosensor cells could occur either due to an accidental entry of several AI molecules into the cell (an unlikely event) or an AI accumulation in the cell caused by the AI binding to LuxR.

It was shown for all tested *luxR* genes that an increase in the regulatory gene dosage causes an increase in the sensitivity of the biosensor to AI. At the same time, an increase in the background luminescence of cells is observed; a similar effect was observed with the *traR A. tumefaciens* regulatory gene [20]. The increase in the background luminosity (Figures 2 and 3) could be explained by a partial degradation of LuxR proteins by proteases with a subsequent AI-independent activation of transcription by their C-terminal fragments. The AI-independent activation of *luxCDABE* and *luxI* promoters by the C-terminal domain of LuxR was shown in [28,32,33]. The *luxR1* protein is not subject to degradation by the Lon protease and does not require GroEL/ES for assembly [22]. It is possible that this, along with the negative regulation of its expression [23], leads to the fact that a change in the dosage of the *luxR1* gene does not affect sensitivity and background luminescence of the biosensor. The cloning of *luxR1* under the non-QS P_{lac} promoter resulted in both an increased sensitivity and an increased background luminescence (Figures 2 and 3). This result suggests that the lack of a gene dosage effect for *luxR1* is more likely associated with P_{luxR1} regulation.

An increase in background luminescence values does not prevent the use of a biosensor with an increased regulatory gene dosage for AI searching in environmental samples. This is contrary to a biosensor with *luxR* under control of an independently inducible promoter—any inductor of it will result in a *luxR* expression induction and a false positive signal of the biosensor. During expeditions to the White and Azov Seas, while using an *E. coli*-based biosensor with an increased dosage of the *luxR A. fischeri* gene, the presence of AI in the intestines of approximately 10% of the fish was detected and three strains of the *Aeromonas* genus, possessing a type I QS system, were isolated. Thus, this biosensor strain is suitable for the HSLs search in complex environments, including content of fish intestine [34]. This suggests its applicability for microflora monitoring on fish farms, in particular to control population density of *A. salmonicida* bacteria, which are pathogenic for commercial fish species [35].

5. Conclusions

In this work, we constructed a set of *E. coli*-based *lux*-biosensors for detection of AI with the use of *luxR* genes from *A. logei* and *A. fischeri.* The AI molecules from the medium could transit through the cell membrane, associate with LuxR transcriptional activator, and induce expression of *luxCDABE* genes, resulting in enhanced luminescence output (Figure 6). The mathematical model predicted an increase in sensitivity to AI of QS system with raised LuxR concentration in the cell. Here it was shown that QS system and, consequently, whole-cell biosensor sensitivity to AI, may be significantly improved by increasing the regulatory gene dosage and its expression level. However, there is an optimum in amount of LuxR in the cell, and an expression of regulatory genes that is too intense could negatively affect the biosensor parameters.

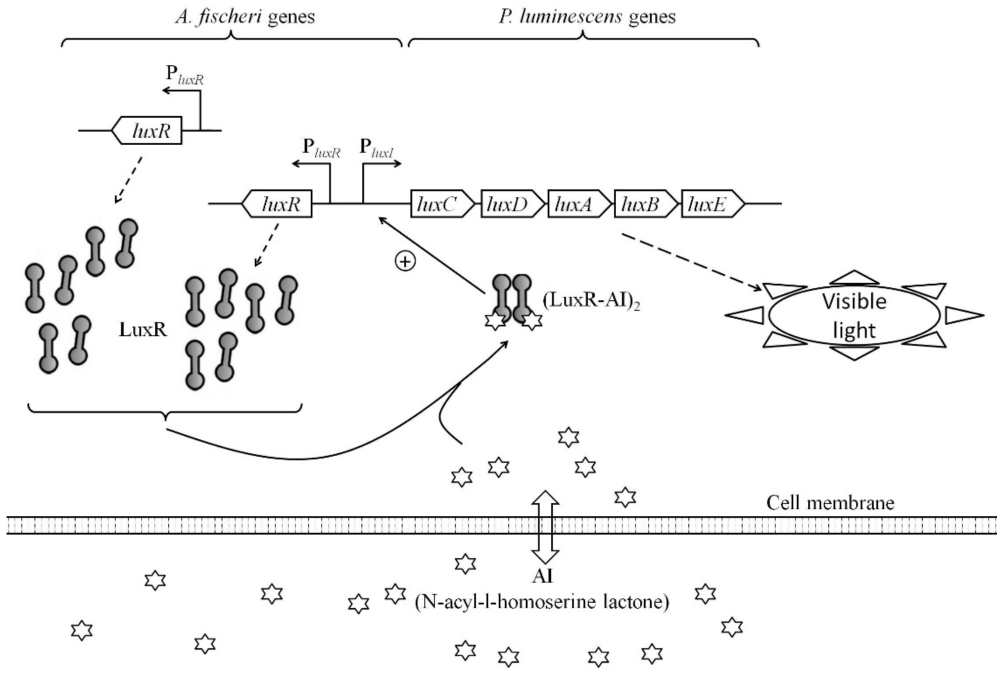

Figure 6. Mechanism of work of the whole-cell *luxR*-based *lux*-biosensor with the enhanced *luxR A. fischeri* dosage.

Supplementary Materials: The following are available online at https://www.mdpi.com/article/10.3390/bios11060166/s1, Figure S1: Scheme of key components of plasmids used in the study with specification of biosensor plasmids names and regulatory gene source bacteria species; Figure S2 The luminescence of biosensor cell culture portions supplemented with 20 µL of environmental samples; Table S1: Oligonucleotides used for cloning promoters into promoter-probe vector and litR under Ptac promoter in this study; Table S2: Samples collected and analyzed with *E. coli* MG1655 pVFR1 pSVRAF biosensor during the expedition to the Azov and to the White Seas.

Author Contributions: Conceptualization, V.C. and I.M.; data curation, S.B., S.K., V.C. and I.M.; investigation, S.B., U.N., E.S. and V.F.; methodology, S.B. and I.M.; project administration, Ilya Manukhov; resources, O.M., S.B. and I.M.; validation, V.F.; visualization, S.B.; writing—original draft, S.B., U.N., E.S. and I.M.; writing—review & editing, S.B., S.K., O.M., V.C. and I.M. All authors have read and agreed to the published version of the manuscript.

Funding: The reported study was funded by RFBR according to the research project № 19-34-90020. The work of I. Manukhov is supported by the Russian Science Foundation under grant 20-16-00088. The work of V. Fomin is supported by Ministry of Science and Higher Education of the Russian Federation, agreement № 075-15-2019-1672 (31.10.2019).

Institutional Review Board Statement: Not applicable.

Informed Consent Statement: Not applicable.

Data Availability Statement: Data is contained within the article or supplementary material.

Acknowledgments: The authors would like to thank Rodygina Polina for the editing of English language and style.

Conflicts of Interest: The authors declare no conflict of interest.

References

1. Nealson, K.H.; Platt, T.; Hastings, J.W. Cellular control of the synthesis and activity of the bacterial luminescent system. *J. Bacteriol.* **1970**, *104*, 313–322. [CrossRef] [PubMed]
2. Eberhard, A.; Burlingame, A.L.; Eberhard, C.; Kenyon, G.L.; Nealson, K.H.; Oppenheimer, N.J. Structural identification of autoinducer of *Photobacterium fischeri* luciferase. *Biochemistry* **1981**, *20*, 2444–2449. [CrossRef]
3. Choi, S.H.; Greenberg, E.P. Genetic evidence for multimerization of LuxR, the transcriptional activator of *Vibrio fischeri* luminescence. *Mol. Mar. Biol. Biotechnol.* **1992**, *1*, 408–413.

4. Fidopiastis, P.M.; Sørum, H.; Ruby, E.G. Cryptic luminescence in the cold-water fish pathogen *Vibrio salmonicida*. *Arch. Microbiol.* **1999**, *171*, 205–209. [CrossRef] [PubMed]
5. Manukhov, I.V.; Khrul'nova, S.A.; Baranova, A.; Zavilgelsky, G.B. Comparative analysis of the *lux* operons in *Aliivibrio logei* KCh1 (a Kamchatka Isolate) and *Aliivibrio salmonicida*. *J. Bacteriol.* **2011**, *193*, 3998–4001. [CrossRef]
6. Konopleva, M.N.; Khrulnova, S.A.; Baranova, A.; Ekimov, L.V.; Bazhenov, S.V.; Goryanin, I.I.; Manukhov, I.V. A combination of *luxR1* and *luxR2* genes activates Pr-promoters of psychrophilic *Aliivibrio logei lux*-operon independently of chaperonin GroEL/ES and protease Lon at high concentrations of autoinducer. *Biochem. Biophys. Res. Commun.* **2016**, *473*, 1158–1162. [CrossRef] [PubMed]
7. Ng, W.-L.; Bassler, B.L. Bacterial Quorum-Sensing Network Architectures. *Annu. Rev. Genet.* **2009**, *43*, 197–222. [CrossRef] [PubMed]
8. Hansen, H.; Purohit, A.A.; Leiros, H.K.S.; Johansen, J.A.; Kellermann, S.J.; Bjelland, A.M.; Willassen, N.P. The autoinducer synthases LuxI and AinS are responsible for temperature-dependent AHL production in the fish pathogen *Aliivibrio salmonicida*. *BMC Microbiol.* **2015**, *15*, 1–13. [CrossRef]
9. Leipert, J.; Treitz, C.; Leippe, M.; Tholey, A. Identification and quantification of N-acyl homoserine lactones involved in bacterial communication by small-scale synthesis of internal dtandards and Matrix-Assisted Laser Desorption/Ionization Mass Spectrometry. *J. Am. Soc. Mass Spectrom.* **2017**, *28*, 2538–2547. [CrossRef]
10. Chan, K.G.; Cheng, H.J.; Chen, J.W.; Yin, W.F.; Ngeow, Y.F. Tandem mass spectrometry detection of quorum sensing activity in multidrug resistant clinical isolate *Acinetobacter baumannii*. *Sci. World J.* **2014**, *2014*, 891041. [CrossRef]
11. Miller, C.; Gilmore, J. Detection of Quorum-Sensing molecules for pathogenic molecules using cell-based and cell-free biosensors. *Antibiotics* **2020**, *9*, 259. [CrossRef]
12. Andersen, J.B.; Heydorn, A.; Hentzer, M.; Eberl, L.; Geisenberger, O.; Christensen, B.B.; Molin, S.; Givskov, M. *gfp*-based N-acyl homoserine-lactone sensor systems for detection of bacterial communication. *Appl. Environ. Microbiol.* **2001**, *67*, 575–585. [CrossRef]
13. Steindler, L.; Venturi, V. Detection of quorum-sensing N-acyl homoserine lactone signal molecules by bacterial biosensors. *FEMS Microbiol. Lett.* **2007**, *266*, 1–9. [CrossRef]
14. Asfour, H. Anti-quorum sensing natural compounds. *J. Microsc. Ultrastruct.* **2018**, *6*, 1. [CrossRef] [PubMed]
15. Kumar, S.; Costantino, V.; Venturi, V.; Steindler, L. Quorum sensing inhibitors from the sea discovered using bacterial N-acyl-homoserine lactone-based biosensors. *Mar. Drugs* **2017**, *15*, 53. [CrossRef]
16. Rai, N.; Rai, R.; Venkatesh, K.V. Quorum Sensing Biosensors. In *Quorum Sensing vs Quorum Quenching: A battle with no end in sight*; Springer India: New Delhi, India, 2015; pp. 171–183. [CrossRef]
17. Pearson, J.P.; Gray, K.M.; Passador, L.; Tucker, K.D.; Eberhard, A.; Iglewski, B.H.; Greenberg, E.P. Structure of the autoinducer required for expression of *Pseudomonas aeruginosa* virulence genes. *Proc. Natl. Acad. Sci. USA* **1994**, *91*, 197–201. [CrossRef] [PubMed]
18. Winson, M.K.; Swift, S.; Fish, L.; Throup, J.P.; JÃ¸rgensen, F.; Chhabra, S.R.; Bycroft, B.W.; Williams, P.; Stewart, G.S.A. Construction and analysis of *luxCDABE* -based plasmid sensors for investigating *N* -acyl homoserine lactone-mediated quorum sensing. *FEMS Microbiol. Lett.* **1998**, *163*, 185–192. [CrossRef]
19. Colton, D.M.; Stabb, E.V.; Hagen, S.J. Modeling analysis of signal sensitivity and specificity by *Vibrio fischeri* LuxR variants. *PLoS ONE* **2015**, *10*, e0126474. [CrossRef]
20. Zhu, J.; Chai, Y.; Zhong, Z.; Li, S.; Winans, S.C. Agrobacterium bioassay strain for ultrasensitive detection of N-acylhomoserine lactone-type Quorum-Sensing molecules: Detection of autoinducers in *Mesorhizobium huakuii*. *Appl. Environ. Microbiol.* **2003**, *69*, 6949–6953. [CrossRef] [PubMed]
21. Fidopiastis, P.M.; Miyamoto, C.M.; Jobling, M.G.; Meighen, E.A.; Ruby, E.G. LitR, a new transcriptional activator in *Vibrio fischeri*, regulates luminescence and symbiotic light organ colonization. *Mol. Microbiol.* **2002**, *45*, 131–143. [CrossRef]
22. Khrulnova, S.A.; Baranova, A.; Bazhenov, S.V.; Goryanin, I.I.; Konopleva, M.N.; Maryshev, I.V.; Salykhova, A.I.; Vasilyeva, A.V.; Manukhov, I.V.; Zavilgelsky, G.B. Lux-operon of the marine psychrophilic bacterium *Aliivibrio logei*: A comparative analysis of the LuxR1/LuxR2 regulatory activity in *Escherichia coli* cells. *Microbiology* **2016**, *162*, 717–724. [CrossRef] [PubMed]
23. Melkina, O.E.; Goryanin, I.I.; Bazhenov, S.V.; Manukhov, I.V.; Zavilgelsky, G.B. Comparative analysis of *Aliivibrio logei luxR1* and *luxR2* genes regulation in *Escherichia coli* cells. *Arch. Microbiol.* **2019**, *201*, 1415–1425. [CrossRef]
24. Zavil'gel'skiĭ, G.B.; Manukhov, I.V. Lon-protease participates in the regulation of transcription of the Lux-operon of *Vibrio fischeri*. *Genetika* **1994**, *30*, 337–341. [PubMed]
25. Guyer, M.S.; Reed, R.R.; Steitz, J.A.; Low, K.B. Identification of a sex-factor-affinity site in *E. coli* as gamma delta. *Cold Spring Harb. Symp. Quant. Biol.* **1981**, *45 Pt 1*, 135–140. [CrossRef]
26. Khrul'nova, S.A.; Manukhov, I.V.; Zavil'gel'skiĭ, G.B. "Quorum sensing" regulation of lux gene expression and the structure of lux operon in marine bacteria *Alivibrio logei*. *Russ. J. Genet.* **2011**, *47*, 1596–1603.
27. Manukhov, I.V.; Kotova, V.I.; Zavil'gel'skiĭ, G.B. Host factors in the regulation of the *Vibrio fischeri lux* operon in *Escherichia coli* cells. *Mikrobiologiia* **2006**, *75*, 525–531. [CrossRef]
28. Manukhov, I.V.; Melkina, O.E.; Goryanin, I.I.; Baranova, A.V.; Zavilgelsky, G.B. The N-terminal domain of *Aliivibrio fischeri* LuxR is a target of the GroEL chaperonin. *J. Bacteriol.* **2010**, *192*, 5549–5551. [CrossRef]

29. Green, M.R.; Sambrook, J. *Molecular Cloning: A Laboratory Manual*, 4th ed.; Cold Spring Harbor Laboratory Press: New York, NY, USA, 2012.
30. Chan, X.Y.; How, K.Y.; Yin, W.F.; Chan, K.G. N-Acyl homoserine lactone-mediated quorum sensing in *Aeromonas veronii* biovar sobria strain 159: Identification of LuxRI homologs. *Front. Cell. Infect. Microbiol.* **2016**, *6*, 7. [CrossRef]
31. Kubitschek, H.E.; Friske, J.A. Determination of bacterial cell volume with the Coulter Counter. *J. Bacteriol.* **1986**, *168*, 1466–1467. [CrossRef] [PubMed]
32. Choi, S.H.; Greenberg, E.P. The C-terminal region of the *Vibrio fischeri* LuxR protein contains an inducer-independent lux gene activating domain. *Proc. Natl. Acad. Sci. USA* **1991**, *88*, 11115–11119. [CrossRef]
33. Mel'kina, O.E.; Manukhov, I.V.; Zavil'gel'skiĭ, G.B. The C-terminal domain of the *Vibrio fischeri* transcription activator LuxR is not essential for degradation by Lon protease. *Mol. Biol. (Mosk.)* **2010**, *44*, 515–519. [CrossRef] [PubMed]
34. Bazhenov, S.V.; Khrulnova, S.A.; Konopleva, M.N.; Manukhov, I.V. Seasonal changes in luminescent intestinal microflora of the fish inhabiting the Bering and Okhotsk seas. *FEMS Microbiol. Lett.* **2019**, *366*. [CrossRef] [PubMed]
35. Egidius, E.; Wiik, R.; Andersen, K. *Vibrio salmonicida* sp. nov., a new fish pathogen. *Int. J. Syst. Bacteriol.* **1986**, *36*, 518–520. [CrossRef]
