# Supplementary material for: Influence of the luxR Regulatory Gene Dosage and Expression Level on the Sensitivity of the Whole-Cell Biosensor to Acyl-Homoserine Lactone
Source: Biosensors (Basel). 2021 May 23;11(6):166. doi: 10.3390/bios11060166 (PMC8224577; doi:10.3390/bios11060166)
Supplement: Supplementary file 1 [file biosensors-11-00166-s001.zip › biosensors-1209223-tex.zip!/Definitions/biosensors-1209223-g002.pdf]

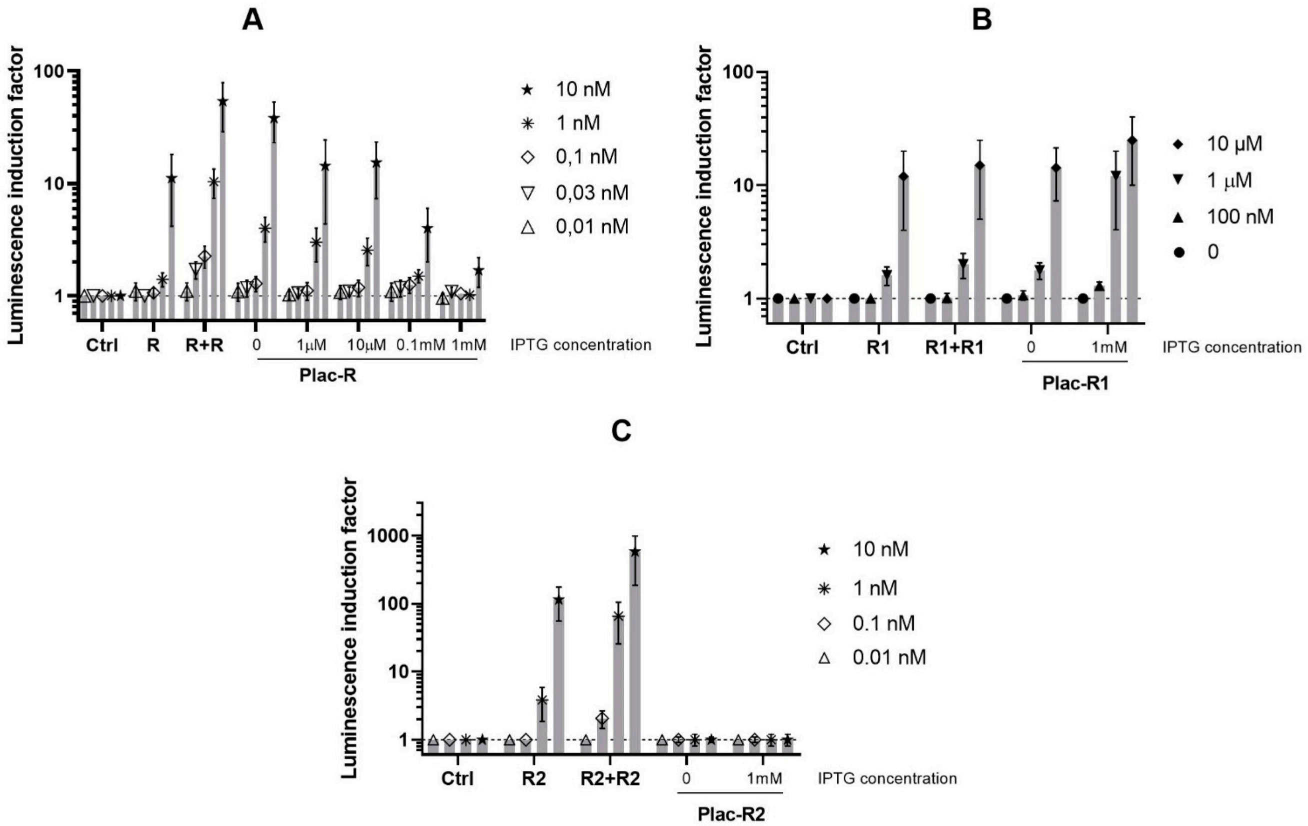
A
Luminescence induction factor
100
10
1
10 nM
1 nM
0,1 nM
0,03 nM
0,01 nM
Ctrl
R
R+R
0
1µM
10µM
0.1mM
1mM
IPTG concentration
Plac-R
B
Luminescence induction factor
100
10
1
10 µM
1 µM
100 nM
0
Ctrl
R1
R1+R1
0
1mM
IPTG concentration
Plac-R1
C
Luminescence induction factor
1000
100
10
1
10 nM
1 nM
0.1 nM
0.01 nM
Ctrl
R2
R2+R2
0
1mM
IPTG concentration
Plac-R2
