# Supplementary material for: Influence of the luxR Regulatory Gene Dosage and Expression Level on the Sensitivity of the Whole-Cell Biosensor to Acyl-Homoserine Lactone
Source: Biosensors (Basel). 2021 May 23;11(6):166. doi: 10.3390/bios11060166 (PMC8224577; doi:10.3390/bios11060166)
Supplement: Supplementary file 1 [file biosensors-11-00166-s001.zip › biosensors-1209223-tex.zip!/Definitions/biosensors-1209223-g003.pdf]

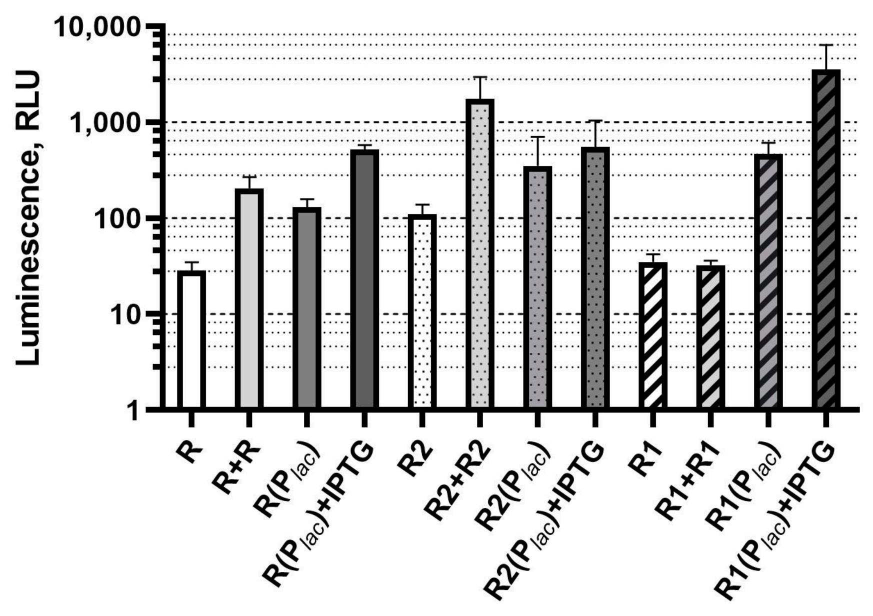

Luminescence, RLU
10,000
1,000
100
10
1
R
R+R
R(P lac)
R(P lac)+IPTG
R2
R2+R2
R2(P lac)
R2(P lac)+IPTG
R1
R1+R1
R1(P lac)
R1(P lac)+IPTG
