# Supplementary material for: Influence of the luxR Regulatory Gene Dosage and Expression Level on the Sensitivity of the Whole-Cell Biosensor to Acyl-Homoserine Lactone
Source: Biosensors (Basel). 2021 May 23;11(6):166. doi: 10.3390/bios11060166 (PMC8224577; doi:10.3390/bios11060166)
Supplement: Supplementary file 1 [file biosensors-11-00166-s001.zip › biosensors-1209223-tex.zip!/Definitions/biosensors-1209223-g004.pdf]

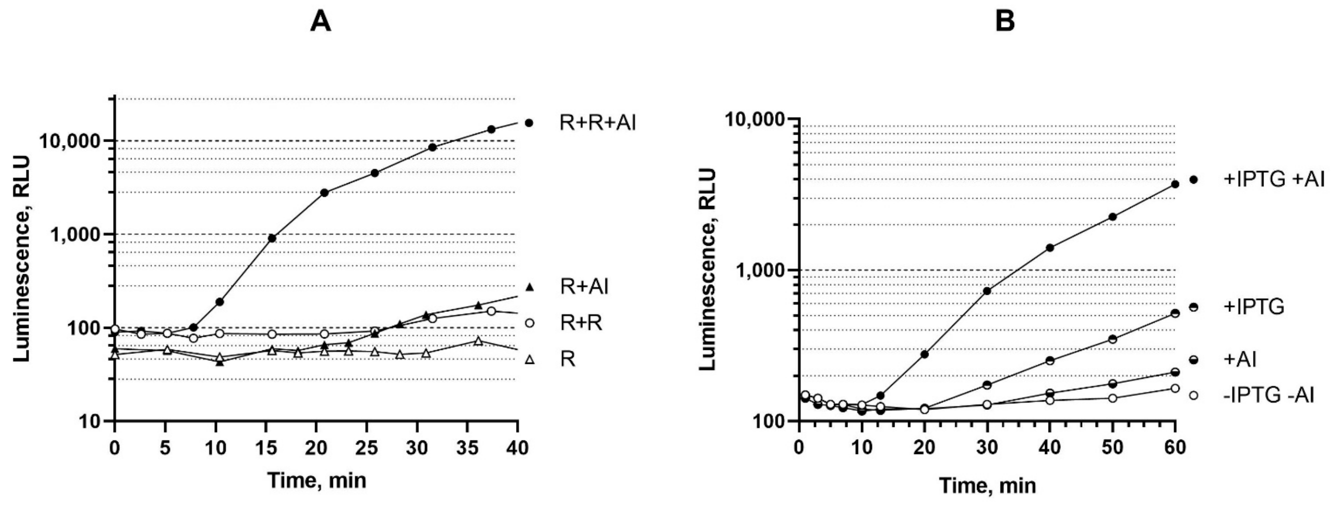
A
B
Luminescence, RLU
10
100
1,000
10,000
0
5
10
15
20
25
30
35
40
Time, min
R+R+AI
R+AI
R+R
R
100
1,000
10,000
0
10
20
30
40
50
60
+IPTG +AI
+IPTG
+AI
-IPTG -AI
