# Supplementary material for: Influence of the luxR Regulatory Gene Dosage and Expression Level on the Sensitivity of the Whole-Cell Biosensor to Acyl-Homoserine Lactone
Source: Biosensors (Basel). 2021 May 23;11(6):166. doi: 10.3390/bios11060166 (PMC8224577; doi:10.3390/bios11060166)
Supplement: Supplementary file 1 [file biosensors-11-00166-s001.zip › biosensors-1209223-tex.zip!/Definitions/biosensors-logo-eps-converted-to.pdf]

biosensors
